# A simple method for exploring adverse drug events in patients with different primary diseases using spontaneous reporting system

**DOI:** 10.1186/s12859-018-2137-y

**Published:** 2018-04-05

**Authors:** Yoshihiro Noguchi, Anri Ueno, Manami Otsubo, Hayato Katsuno, Ikuto Sugita, Yuta Kanematsu, Aki Yoshida, Hiroki Esaki, Tomoya Tachi, Hitomi Teramachi

**Affiliations:** Laboratory of Clinical Pharmacy, Gifu Pharmaceutical University, 1-25-4,Daigakunishi, Gifu, 501-1196 Japan

## Abstract

**Background:**

Patient background (e.g. age, sex, and primary disease) is an important factor to consider when monitoring adverse drug events (ADEs) for the purpose of pharmacovigilance. However, in disproportionality methods, when additional factors are considered, the number of combinations that have to be computed increases, and it becomes very difficult to explore the whole spontaneous reporting system (SRS). Since the signals need to be detected quickly in pharmacovigilance, a simple exploration method is required. Although association rule mining (AR) is commonly used for the analysis of large data, its application to pharmacovigilance is rare and there are almost no studies comparing AR with conventional signal detection methods.

**Methods:**

In this study, in order to establish a simple method to explore ADEs in patients with kidney or liver injury as a background disease, the AR and proportional reporting ratio (PRR) signal detection methods were compared. We used oral medicine SRS data from the Japanese Adverse Drug Event Report database (JADER), and used AR as the proposed search method and PRR as the conventional method for comparison. “Rule count ≥ 3”, “min lift value > 1”, and “min conviction value > 1” were used as the AR detection criteria, and the PRR detection criteria were “Rule count ≥3”, “PRR ≥ 2”, and “χ^2^ ≥ 4”.

**Results:**

In patients with kidney injury, the AR method had a sensitivity of 99.58%, specificity of 94.99%, and Youden’s index of 0.946, while in patients with liver injury, the sensitivity, specificity, and Youden’s index were 99.57%, 94.87%, and 0.944, respectively. Additionally, the lift value and the strength of the signal were positively correlated.

**Conclusions:**

It was suggested that computation using AR might be simple with the detection power equivalent to that of the conventional signal detection method as PRR. In addition, AR can theoretically be applicable to SRS other than JADER. Therefore, complicated conditions (patient’s background etc.) that must take factors other than the ADE into consideration can be easily explored by selecting the AR as the first screening for ADE exploration in pharmacovigilance using SRS.

## Background

Recently, due to advances in information technology, large data have begun to be utilized in many fields. Among them is the field of medical monitoring, where numerous risk assessments of drugs have been reported using spontaneous reporting system (SRS), which are based on spontaneous reports of drug-adverse event (AE) pair accumulated and published by regulatory authorities [1–5].

Various risk assessment methods exist for evaluating adverse drug events (ADEs) based on SRS data, including those based on the proportional reporting ratio (PRR) [6], which is used by the Medicines and Healthcare Products Regulatory Agency (MHRA), and the reporting odds ratio (ROR) [7], which is used by the Netherlands Pharmacovigilance Center Lareb. In addition, a Bayesian Confidence Propagation Neural Network (BCPNN) -based method [8] is used by the World Health Organization (WHO), and a Gamma-Poisson Shrinker (GPS) -based method [9] is used by the United States Food and Drug Administration (FDA). These methods are all signal detection methods used in disproportionality analysis, based on the principle of inequality, focusing on differences in the ratio of the number of reported drug-AE pairs.

If there is no causal relationship between the drug of interest and the AE, the reporting ratio should be approximately the same as the average reporting ratio of other medicines overall. If the reporting ratio of the drug of interest is significantly higher than the average reporting ratio, it is indicative of an ADE, suggesting a causal relationship between the drug and AE [10].

Since the PRR and the ROR are easy to calculate, it is possible to detect an ADE at an early stage, and since these methods are sensitive, there is little risk of missing a true signal.

**Fig. 1:**
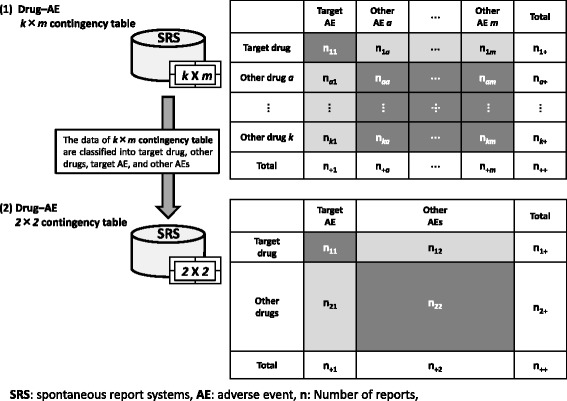
Create the drug–AE *k* × m contingency table to *2* × *2* contingency table for signal detection

These methods use data from an SRS database to create “drug–AE pairs *k* × *m* contingency table”. In a typical “drug–AE pairs *2* × *2* contingency table” the data are classified into target drug, other drugs, target AE, and other AEs, and the table is used to calculate a risk evaluation index as shown in Fig. 1.

When evaluating ADEs, patient background (e.g. age, sex and primary disease) is another important factor that should be taken into consideration. However, in order to consider additional factors, the drug–AE pairs *2* × *2* contingency table must be prepared from a database that extracts data for each factor, and signal indices must be calculated. As a result, the number of combinations becomes enormous, and it is very difficult to implement the calculations efficiently. Thus, a simple signal detection method that allows other factors to be easily considered is urgently needed.

In large data analysis, association rule mining (AR) is aimed at “enumerating interesting patterns hidden in the database” [11–13]. Several analysis methods using AR in pharmacovigilance have recently been proposed [14–17], but there are few reports comparing AR with conventional signal detection methods [17].

Therefore, in this study, in order to establish a simple method for exploring ADEs in patients with kidney injury or liver injury as a primary disease, signal detection using AR and PRR methods was compared.

## Methods

We used the SRS dataset from the 1st quarter of 2004 to the 4th quarter of 2015 from the Japanese Adverse Drug Event Report database (JADER). The JADER was downloaded from Pharmaceuticals and Medical Devices Agency and composed of four tables as follows: DEMO table (with information on gender, age, and weight), DRUG table (with information on suspect drug and concomitant drug), REAC table (with information on AE and outcome), and HIST table (containing medical history of primary diseases and secondary diseases) [18]. Duplicate data and data for non-oral medications were removed from the JADER, and the remaining 184,917 cases were analyzed.

**Fig. 2:**
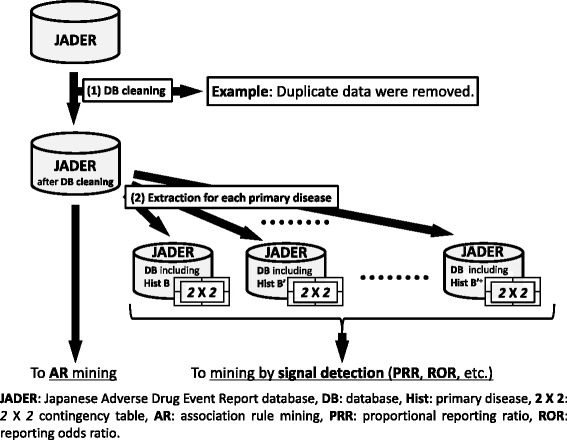
Database processing for AR mining and mining by signal detection considered about primary disease

Kidney injury and liver injury can affect drug metabolism, so these primary diseases were considered. The AEs registered in the JADER are represented using the preferred terms (PTs) from the Medical Dictionary for Regulatory Activities (MedDRA). We extracted all of the data for each PT included in the standardized MedDRA Queries (SMQ) for kidney injury and liver injury, which are standard search formulae for MedDRA. The methods used to extract the data from the JADER are illustrated in Fig. 2.

**Fig. 3:**
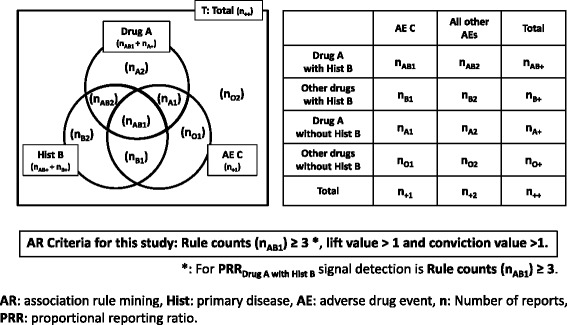
The calculation of AR for the Venn diagram and the drug–AE pairs *4* × *2* contingency table

In this study, we used “lift” and “conviction” as the detection criteria for searching the association rule “A ∩ B → C”, where A is the drug, B is the primary disease, and C is the AE. The calculation methods are shown in Fig. 3 and formula (1) and (2).

1 $$ {\displaystyle \begin{array}{c}\mathrm{lift}\ \left(\mathrm{A}\cap \mathrm{B}\to \mathrm{C}\right)=\mathrm{confidence}\ \left(\mathrm{A}\cap \mathrm{B}\to \mathrm{C}\right)/\mathrm{support}\ \left(\mathrm{C}\right)\\ {}=\left({\mathrm{n}}_{\mathrm{AB}1}/{\mathrm{n}}_{\mathrm{AB}+}\right)/\left({\mathrm{n}}_{+1}/{\mathrm{n}}_{++}\right)\end{array}} $$

2 $$ {\displaystyle \begin{array}{l}\begin{array}{l}\mathrm{conviction}\ \left(\mathrm{A}\cap \mathrm{B}\to \mathrm{C}\right)\\ {}\kern8em =\Big(1\hbox{--} \mathrm{Support}\ \left(\mathrm{C}\right)/\left(1\hbox{--} \mathrm{C}\mathrm{onfidence}\ \left(\mathrm{A}\cap \mathrm{B}\to \mathrm{C}\right)\ \right)\end{array}\kern1.25em \\ {}\kern7.75em =\left(1\hbox{--} {\mathrm{n}}_{+1}/{\mathrm{n}}_{++}\right)/\left(1\hbox{--} {\mathrm{n}}_{\mathrm{AB}1}/{\mathrm{n}}_{\mathrm{AB}+}\right)\end{array}} $$

“Lift” is an index that indicates the relative magnitude of the probability of observing C under the condition of A ∩ B, compared to the overall probability of observing C. When the lift value is 1, the two events A ∩ B and C are independent of each other. When the lift value is greater than 1, the two events A ∩ B and C are not independent, and the higher the value, the greater the relevance of the interaction [19].

On the other hand, “conviction” is an indicator that evaluates whether or not the rule makes a wrong prediction, paying particular attention to the exclusion event of the conclusion part of the obtained rule. If the conviction value is large, it is less likely that the conclusion C is not true for the premise A ∩ B [20].

In general, lift > 1 is used as the detection standard for the AR method, but conviction > 1 was also used in this study. Furthermore, “Rule count (n_AB 1_) ≥ 3” was also used according to the detection criteria of the PRR method for comparison.

**Fig. 4:**
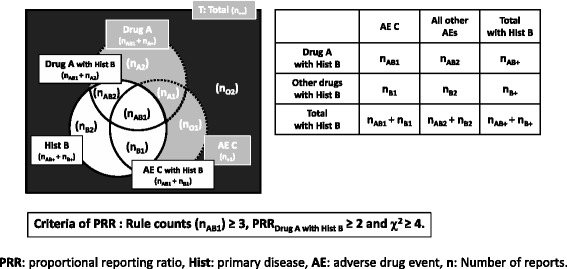
The calculation of PRR_Drug A with Hist B_ for the Venn diagram and the drug–AE *2* × *2* contingency table

In addition, in order to verify the accuracy of the signal detected by the AR method, we compared it with the signal detected using the PRR method, which is a conventional signal detection method. As shown in Figs. 2 and 4 and formula (3) and (4), the PRR signal value is calculated from “the drug–AE pairs *2* × *2* contingency table” for each case. In the data set, Drug A, Hist B and AE C can be represented by the Venn diagram shown in Fig. 3, but only the part limited to Hist B shown in Fig. 4 is used for calculation of PRR value. The calculation method of PRR is similar to risk ratio which is a general statistical index. According to the criteria of MHRA, the detection standard for the PRR method is “Rule count (n_AB 1_) ≥ 3”, “PRR ≥ 2”, and “χ^2^ ≥ 4” [6].

Since there were no simulation data in this study, there are no complete data of true risk. Therefore assuming that the signal detected by PRR is true risk, the accuracy of signal detection using the AR method was examined using sensitivity, specificity, Youden’s index, positive predictive value (PPV), negative predictive value (NPV), receiver operating characteristic (ROC) curve and area under the ROC curve (AUC). However, based on the assumption, the cutoff value was not calculated in this study.

Furthermore, the correlation which was investigated using a single regression line between the lift value of the AR method and the signal intensity of the PRR method was also examined (Fig. 4). When examining this correlation, the signal intensity of the PRR is expressed as “log PRR + log χ^2^”, as proposed by Takagi et al. [15].

3 $$ {\mathrm{PRR}}_{\mathrm{Drug}\ \mathrm{A}\ \mathrm{with}\ \mathrm{Hist}\ \mathrm{B}}=\left({\mathrm{n}}_{\mathrm{AB}1}/{\mathrm{n}}_{\mathrm{AB}+}\right)/\left({\mathrm{n}}_{\mathrm{B}1}/{\mathrm{n}}_{\mathrm{B}+}\right) $$

4 $$ {\upchi}^2=\left({\mathrm{n}}_{\mathrm{AB}+}+{\mathrm{n}}_{\mathrm{B}+}\right){\left\{\left|{\mathrm{n}}_{\mathrm{AB}1}{\mathrm{n}}_{\mathrm{B}2}-{\mathrm{n}}_{\mathrm{AB}2}\;{\mathrm{n}}_{\mathrm{B}1}\right|-\left({\mathrm{n}}_{\mathrm{AB}+}+{\mathrm{n}}_{\mathrm{B}+}\right)/2\right\}}^2/\left\{\right({\mathrm{n}}_{\mathrm{AB}+}{\mathrm{n}}_{\mathrm{B}+}\left({\mathrm{n}}_{\mathrm{AB}1}+{\mathrm{n}}_{\mathrm{B}1}\right)\;\left({\mathrm{n}}_{\mathrm{AB}2}+{\mathrm{n}}_{\mathrm{B}2}\right)\Big\} $$

Data management and analyses were performed using Visual Mining Studio software (version 8.1; Mathematical Systems, Inc. Tokyo, Japan). Drawing the ROC curve and AUC calculation were performed using JMP 11.2.0 (SAS Institute Inc.)

## Results

Among all the cases analyzed (184,917 cases), there were 18,252 cases (24,463 drug–ADE pairs) of kidney injury, and 23,183 cases (23,460 drug–ADE pairs) of liver injury. The number of signals detected for the ADEs using the PRR was 2371 drug–ADE pairs for kidney injury, and 2303 pairs for liver injury.

Table 1 shows the signal detection power of AR for each primary diseases. For kidney injury, the sensitivity was 99.58%, specificity was 94.99%, Youden’s index was 0.946, PPV was 68.08%, and NPV was 99.95%. For liver injury, the sensitivity was 99.57%, specificity was 94.87%, Youden’s index was 0.944, PPV was 67.88%, and NPV was 99.95%.

**Table 1:** Ability of AR to detect signals for primary diseases

Primary diseases	Sensitivity (%)	Specificity (%)	Youden’s index	PPV (%)	NPV (%)
Kidney injury	99.58	94.99	0.946	68.08	99.95
Liver injury	99.57	94.87	0.944	67.88	99.95

*AR* association rule mining, *PPV* positive predictive value, *NPV* negative predictive value

**Fig. 5:**
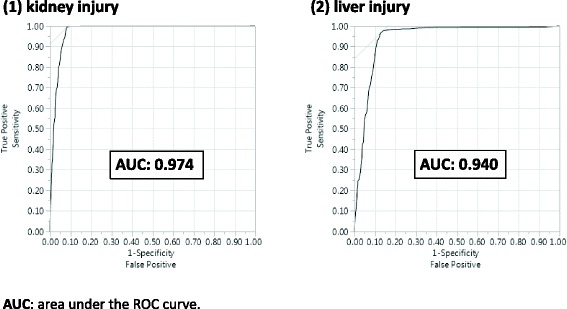
The ROC curve and AUCs for each primary diseases

**Fig. 6:**
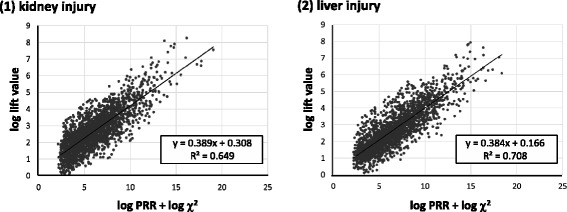
Relation between log lift value and log PRR + log χ^2^ for each primary diseases

Figure 5 shows the ROC curve. For kidney injury, the AUC was 0.974, and for liver injury, the AUC was 0.940. Figure 6 shows the correlation between the lift value and PRR signal intensity. The decision coefficient (R^2^) = 0.649 for kidney injury and R^2^ = 0.708 for liver injury.

## Discussion

Although there are several reports of previous studies on the exploration of ADEs using SRS, reports that consider factors other than the drug–AE pairs, such as the primary disease affecting the patient, are limited. The reason for this is that in disproportionality methods, considering additional factors requires an enormous number of combinations to be taken into account when signal detection is performed and the respective risk indicators are calculated. Therefore, considering additional factors seems to be impractical as an exploration method.

A potential solution to this issue would be to utilize AR. However, although AR is often used to efficiently analyze large data, there are only a few examples of it being used in the medical field, especially in SRS analysis [14–17]. Signal detection using AR has already been validated as an effective method for the initial identification of “multi-item ADEs” in a study by Harpaz et al. [14]. Furthermore, although information on primary diseases was not included similar to this study, the use of AR in drug–AE pairs was compared with the conventional signal detection method by Wang et al. [17]. Currently, signal detection using AR is not used in pharmacovigilance at regulatory authorities, but is considered very useful for performing complicated analysis considering the patient’s background as primary disease.

Therefore, in this study, kidney injury or liver injury as primary disease was considered in addition to the drug–AE pairs, and signal detection was performed using AR method. The conventional PRR method was also used, and the signal detection powers of each method were compared.

In this study, the detection criteria for AR were n_AB 1_ ≥ 3, lift > 1, and conviction > 1. For kidney or liver injury, in the AR method, both sensitivities were greater than 99%, and both specificities were greater than 94%. The same detection results were also obtained using the PRR method, indicating that the detection powers of the AR and PRR methods are similar. In addition, the AUC was 0.974 for kidney injury and 0.940 for liver injury. These high values suggest that the AR method is also highly accurate.

However, the NPV was greater than 99.9% for both kidney and liver injury, but the PPV was only 68.08% for kidney injury and 67.88% for liver injury.

These results suggest that the AR method may have detected signals that could not be detected by the conventional PRR method. However, unfortunately, we cannot prove our hypothesis, because we did not have “the true risk”. The true risk dataset containing “unknown AEs” does not exist.

Because SRS is the result of voluntary reporting and is influenced by reporting bias including underreporting, and the value of the signal easily changes depending on the timing of the analysis, signal detection is not necessarily the true risk, but is limited to the hypothesis of risk. In other words, PRR signals and AR signals are limited to the hypothesis of risk, but they are not the true risk.

The detected signals are hypotheses to be clinically noticed until pharmacologic verification is completed. For the AR method to be an alternative to the PRR method, a correlation with the magnitude of the signal value is desirable.

The magnitude of the lift values as AR signals and the PRR signals intensity are positively correlated. Thus, the correlation of the signal values of each method also makes the AR method easy to use for pharmacovigilance.

The conventional PRR method involves extracting data for each of the primary diseases, as shown in Figs. 1, 2 and 4, and constructing a *2* × *2* drug–AE *k* × *m* table. If a similar calculation method as shown in Figs. 2 and 3 that simply creates combinations from the database was used for AR method, the number of the combinations considered would be enormous and it would be difficult to calculate within a realistic time, even if AR method is used.

However, in the AR method, the “apriori algorithm” can be used to reduce the number of calculations. The apriori algorithm is based on the principle that “support of a certain item set is always less than or equal to support of its partial item set” [12]. Therefore, it is unnecessary to calculate the risk index for all combinations, which is required in the conventional method.

In this study, the AR method proposed also requires verification for primary diseases other than kidney injury and liver injury. However, it was suggested that computation using the “apriori algorithm” of AR method might be simple with the detection power equivalent to that of the conventional PRR method.

## Conclusion

The use of post-marketing drugs is complicated, and unlike clinical trials, background factors of patients are diverse. In addition, the frequency of occurrence of ADEs in clinical trial is not known, and there are ADEs that occur over a period longer than the duration of the clinical trial.

SRS analysis using signal detection enables the exploration of unknown ADEs not found in clinical trials and safety assessments in specific populations. It is possible to evaluate safety reflecting the actual clinical use situation. In addition, SRS has played a major role in pharmaepidemiological studies centered on drug safety assessment.

The PRR, which is a conventional signal detection method, is suggestive of ADEs; a similar detection tendency was observed for the AR method. Then, the signal value should be calculated quickly for pharmacological and clinical research. Therefore, in order to reveal the true risk, further pharmacological and clinical research is needed based on the hypothesis obtained. If the method of signal detection is simplified, it will be possible to detect more unknown ADE at an early stage. This is considered important for conducting pharmacological and clinical verification.

In this study, it was suggested that computation using AR method might be simple with the detection power equivalent to that of the conventional signal detection method. In addition, AR method can theoretically be applicable to SRS other than JADER. Therefore, complicated conditions (patient’s background etc.) that must take factors other than the AE–drug pairs into consideration can be easily explored by selecting the AR method as the first screening in pharmacovigilance using SRS.

## Abbreviations

ADE: Adverse drug event
AE: Adverse event
AR: Association rule mining
AUC: Area under the ROC curve
BCPNN: Bayesian Confidence Propagation Neural Network
FDA: Food and Drug Administration
GPS: Gamma-Poisson Shrinker
JADER: the Japanese Adverse Drug Event Report database
MedDRA: Medical Dictionary for Regulatory Activities
MHRA: Medicines and Healthcare Products Regulatory Agency
NPV: Negative predictive value
PPV: positive predictive value
PRR: Proportional reporting ratio
PT: Preferred term
ROC: Receiver operating characteristic
ROR: Reporting odds ratio
SMQ: Standardized MedDRA Queries
SRS: Spontaneous reporting system
WHO: World Health Organization
